# Diagnosis and treatment outcomes of urethritis-like symptoms in young males: a retrospective cohort study

**DOI:** 10.1038/s41598-023-44733-z

**Published:** 2023-10-14

**Authors:** Yi-Ting Hsu, Tzu-Yu Chuang, Jui Chang Hsiao, Weiming Cheng

**Affiliations:** 1 Division of Urology, Department of Surgery, Taipei City Hospital, Renai Branch, Taipei, Taiwan; 2 Division of Urology, Department of Surgery, Taipei City Hospital, Zhongxiao Branch, Taipei, Taiwan; 3 Department of Clinical Laboratory, Taipei City Hospital, Zhongxiao Branch, Taipei, Taiwan; 4 Department of Urology, Faculty of Medicine, College of Medicine, National Yang Ming Chiao Tung University, Taipei, Taiwan

**Keywords:** Infectious diseases, Urogenital diseases, Disease prevention, Prognosis, Public health

## Abstract

The study evaluated the prevalence of gonorrhoea and chlamydia infections and find out other non-infectious diseases in sexually active young males with urethritis-like symptoms and their treatment outcomes. We retrospectively reviewed the young adult males (aged 20–50 years) who visited our clinic with urethritis symptoms from March 2019 to April 2022. All patients underwent urinalysis, urine culture, and urinary polymerase chain reaction (PCR) testing for gonorrhoea and chlamydia. Student’s t-test and Pearson’s chi-square test were used to compare the differences between the triple-negative group (i.e., negative results in urinalysis, urine culture, and urinary PCR) and the any-positive group. Logistic regression analyses were used to evaluate the predictive factors for positive PCR results for gonorrhoea or chlamydia in patients with negative urinalysis and urine culture. Of the 365 participants with urethritis-like symptoms, 139 patients were diagnosed of gonococcal or chlamydia urethritis. Among the 202 patients with negative urinalysis and urine culture, 60 patients were diagnosed with gonorrhoea or chlamydia using PCR. Urethral discharge was an independent predictor. 142 patients with triple negative results were attributed to other non-infectious diseases. Empirical antibiotic treatment is recommended for patients with urethritis symptoms showing positive or negative urinalysis results but with urethral discharge.

## Introduction

The symptoms of urethritis in males include acute-onset dysuria or increased urinary frequency and urgency. Some patients may present with purulent discharge from the urethral meatus, dyspareunia, haematuria, and haematospermia. Among young males, urethritis is most commonly caused by sexually transmitted infections (STIs), including *Neisseria gonorrhoeae* and *Chlamydia trachomatis* infections. It could categorized as gonococcal urethritis, non-gonococcal urethritis (NGU), or non-chlamydial non-gonococcal urethritis based on the results of urine culture and multiplex polymerase chain reaction (PCR). Possible pathogens in cases of non-chlamydial non-gonococcal urethritis include *Mycoplasma genitalium, Ureaplasma urealyticum,* and *Trichomonas vaginalis*^1,2^, while other non-infectious diseases may also result in symptoms similar to those of urethritis.

The present study aimed to investigate the prevalence of gonorrhoea and chlamydia infections and find out other non-infectious diseases in patients presenting with urethritis symptoms. Urinalysis and urine culture are the initial examinations; however, diagnosis of urethritis based on urinalysis and urine culture is not always feasible in clinical practice. One study has shown that most of those with potential STIs were commonly negative for urinalysis and urine culture^3^. The performance of urinary PCR for the detection of gonococcal and chlamydial infections is recommended as screening or diagnostic tests since its high sensitivity^4,5^. Although PCR can yield more accurate diagnoses of urethritis, it is costly and not always available in every clinic. Therefore, it is important to find out clinic-predictive factors of gonococcal or chlamydial urethritis in patients with sterilised urine. Moreover, few studies have reported the possible aetiologies and treatment outcomes of young males with urethritis-like symptoms. Young males sometimes experienced persistent or intermittent urethral pain in the absence of proven infection. Diagnosis is therefore mainly based on exclusion^6,7^. In the present study, we retrospectively collected and analyzed data on the clinical presentations, possible aetiologies, treatment, and prognosis of young adult males with symptoms of urethritis. We also demonstrated a flow chart for quick management on the patient’s first visit. The findings of the present study will be helpful for urologists in treating young patients with symptoms of urethritis.

### Ethics statement

All procedures were in accordance with the ethics committee and with the principles of the Declaration of Helsinki. This study was approved by the Institutional Review Board of Taipei City Hospital (TCHIRB-11106012-E), and the need for informed consent was waived due to the retrospective nature of the study.

## Methods

### Study population and design

We retrospectively reviewed the data of consecutive male patients who visited our clinic from March 2019 to April 2022. Inclusion criteria were young adult males (aged 20–50 years) and presentation of urethritis-like symptoms at the first visit. Those with congenital or structural urogenital anomalies, and those who did not receive follow-up for at least one month were excluded. Participants were classified into two groups based on test results. Those patients with triple-negative findings (i.e., negative results in urinalysis, urine culture, and urinary PCR) were defined as the triple-negative group, while those with positive results in any of the three tests were defined as the any-positive group. We compared the differences between these two groups and investigated the tentative diagnosis and therapy in the triple-negative group. The predictive factors of positive PCR tests for patients with negative urinalysis and urine culture results were also analyzed.

### Data collection

Urethritis-like symptoms included dysuria, increased urination frequency, and urethral discharge. Demographic data, including age at presentation, comorbidities, prior unprotected sexual intercourse, reports of urethral discharge, associated symptoms and treatment outcome were collected and analysed. Considering their symptoms and that they were sexually active at a young age, all patients underwent urinalysis, urine culture, and urinary PCR for *N. gonorrhoeae* and *C. trachomatis* at the first visit. Our institution uses the BD MAX™ CT/GC/TV assay as a PCR test. The assay is an all-in-one automated DNA extraction and real-time PCR test for the direct, qualitative detection of DNA from chlamydia, gonorrhea, and trichomoniasis with a single specimen collection. The assay may be used for detection of DNA in male urine specimens and could provide high sensitivity and accuracy^8^. Treatments were administered based on the initial urinalysis results and clinical judgment of the urologists. All patients were asked to return to our clinic after one week of treatment for repeated urinalysis. The treatment was changed depending on the patient’s response to the initial treatment or positive results in urine culture or urinary PCR. HIV and syphilis tests were recommended for those with positive results of urinary PCR. For patients showing triple-negative findings, the urologists could treat these patients based on their clinical judgement. Further examination would be arranged and follow-up data for symptom resolution were collected one month later.

### Sample size calculation

Based on the primary outcome, the sample size was powered on a common symptom urethral discharge in men with urethritis. Bellinato et al. reported urethral discharge in patients with PCR-identified urethritis with similar patient population^9^. Based on this comparable research, we estimated that the triple-negative group would have 50% incidence of urethral discharge and any-positive group would have 80% incidence of urethral discharge. With an α level of 0.05 and power of 0.90, the required sample size was 51 men in each group. To adjust for an approximate loss-to-follow-up rate of 20%. We aimed to review at least 128 men total.

### Statistical analysis

All data were expressed as number (percentage) or mean ± standard deviation. Student’s t-test and Pearson’s chi-square test were used to compare the differences in continuous and categorical parameters between participants in the triple-negative and any-positive groups. A Venn diagram was used to demonstrate the test patterns in patients with urethritis-like symptoms. Univariate and multivariate logistic regression analyses were used to evaluate the predictive factors for positive gonorrhoea or chlamydia PCR results in patients with negative urinalysis and urine culture results. Statistical analyses were performed using IBM SPSS Statistics for Mac ver. 25 (IBM Corp., Armonk, NY, USA). All statistical analyses were performed using 2-sided tests and a p value < 0.05 was considered statistically significant.

### Ethics approval and consent to participate

The study protocol for this research project has been approved by the Institutional Review Board of Taipei City Hospital, and it conforms to the provisions of the Declaration of Helsinki. The requirement for informed consent was waived by the Ethics Committee of (Institutional Review Board of Taipei City Hospital, Approval No. TCHIRB-11106012-E) because of the retrospective nature of the study.

## Results

A total of 407 young adult males with symptoms of urethritis were included in the study from March 2019 to April 2022. 42 participants were excluded, including 3 men with congenital or structural urogenital anomalies and 39 men who received follow-up less than one month. Of the final 365 study participants, 142 (38.9%) patients were classified into the triple-negative group and 223 (61.1%) patients were classified into the any-positive group. Table 1 shows the demographic characteristics of the patients, including the age at diagnosis, presence of comorbidities, and associated symptoms. Unprotected sexual intercourse in the previous two weeks was only reported in 97 (26.6%) patients. Urethral discharge was reported in 195 (53.4%) patients. Besides urethral discharge and dysuria, painful sensation at the scrotum was the most common associated symptom observed in 46 (12.6%) patients. Persistent symptoms were observed in 22 (6.0%) patients after initial empirical therapy. Patients of the triple-negative group were significantly older (37.0 ± 9.5 years vs. 34.0 ± 8.2 years, p = 0.001) but had less frequent reports of urethral discharge (8.5% vs. 82.1%, p < 0.001) and less symptom persistence (0% vs. 9.9%, p < 0.001) than those of the any-positive group. The incidences of prior unprotected sexual intercourse, comorbidities, and other associated symptoms were similar between the two groups.

**Table 1:** Characteristics of patients with urethritis symptoms and differences between patients in the any-positive and triple-negative groups.

Characteristics	Total patients (n = 365)	Triple-negative group (n = 142)	Any-positive group (n = 223)	p value
Age, years (SD)	35.2 (8.8)	37.0 (9.5)	34.0 (8.2)	0.001
Comorbidity, no. (%)	11 (3.0%)	1 (0.7%)	10 (4.5%)	0.056
Unprotected sex, no. (%)	97 (26.6%)	34 (23.9%)	63 (28.3%)	0.364
Discharge, no. (%)	195 (53.4%)	12 (8.5%)	183 (82.1%)	< 0.001
Scrotal pain, no. (%)	46 (12.6%)	20 (14.1%)	26 (11.7%)	0.520
Haematuria: no. (%)	13 (3.6%)	4 (2.8%)	9 (4.0%)	0.773
Haematospermia, no. (%)	9 (2.5%)	4 (2.8%)	5 (2.2%)	0.740
Erectile dysfunction, no. (%)	8 (2.2%)	3 (2.1%)	5 (2.2%)	0.934
Premature ejaculation, no. (%)	2 (0.5%)	0 (0.0%)	2 (0.9%)	0.523
Perineal pain, no. (%)	5 (1.4%)	0 (0.0%)	5 (2.2%)	0.161
Persistent symptoms, no. (%)	22 (6%)	0 (0.0%)	22 (9.9%)	< 0.001

Triple-negative group = negative results in urinalysis and urine culture and gonorrhoea/chlamydia PCR; any-positive group = positive results in urinalysis, urine culture, or gonorrhoea/chlamydia PCR.

**Figure 1:**
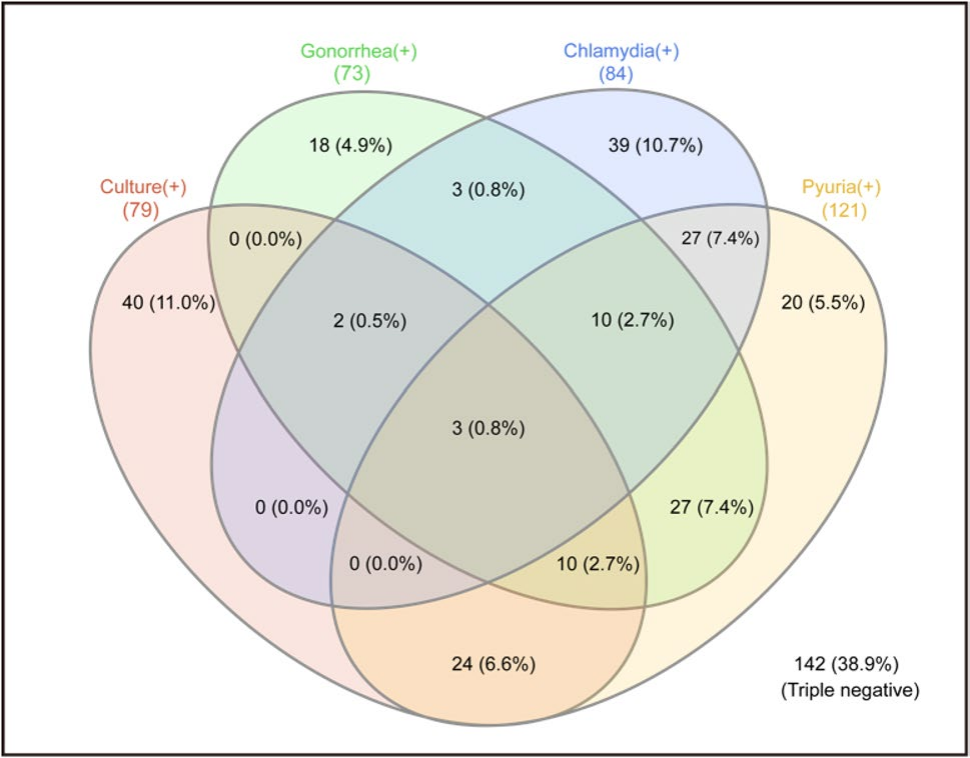
Venn diagram of the 365 genitourinary outpatients with urethritis-like symptoms.

Figure 1 shows a Venn diagram of the results of urinalysis, urine culture, and PCR in 365 patients with symptoms of urethritis. A total of 139 (38.1%) patients were diagnosed of gonococcal or chlamydia urethritis, including 55 (15.1%) with gonococcal urethritis solely and 66 (18.1%) with chlamydia urethritis solely. 18 (4.9%) patients were diagnosed of both pathogens. 121 patients presented with pyuria and 79 patients had positive urine culture results. Among the 202 patients with negative urinalysis and urine culture results, 60 (29.7%) patients were diagnosed with gonorrhoea or chlamydia using a PCR test. 142 (38.9%) patients with triple negative results were attributed to other non-infectious diseases.

Among the 121 patients showing pyuria on urinalysis, 37 (30.6%) showed positive urine culture results, but only 13 (10.7%) were confirmed to have gonococcal infection on urine culture. Microorganisms included Enterococcus faecalis and Klebsiella pneumoniae were detected in 24 (19.8%) patients, but the colony numbers were not sufficient to confirm to be a pathogen. PCR results confirmed gonococcal urethritis in 37 (30.6%) patients, chlamydial urethritis in 37 (30.6%) patients and co-infection in 10 (8.3%) patients. Among the 244 patients without pyuria on urinalysis, 42 (17.2%) patients showed positive urine culture results, but only 2 (0.8%) were confirmed to have gonococcal infection on urine culture. Other microorganisms of the rest 40 (16.4%) were contaminated human skin microorganisms. PCR results confirmed gonococcal urethritis in 21 (8.6%) patients, chlamydial urethritis in 42 (17.2%) patients and co-infection in 3 (1.2%) patients. The *N. gonorrhoeae* isolates in all 15 cases diagnosed by urine culture were susceptible to cefoxitin, cefotaxime, and ceftriaxone.

Among the 202 patients showing negative results in urinalysis and urine culture, 60 (29.7%) patients were diagnosed as having gonorrhoea or chlamydia infection through PCR. In other words, 60 (43.2%) patients out of 139 patients with gonococcal and chlamydia urethritis presented with negative urinalysis and urine culture tests. In the univariate and multivariate logistic regression analyses, the presence of discharge was an independent predictor for a diagnosis of gonorrhoea or chlamydia by PCR (odds ratio, 32.669; 95% confidence interval 13.67–78.09; p < 0.001), after adjustment for age and history of unprotected sexual intercourse (Table 2).

**Table 2:** Predictive factors of positive gonorrhoea or chlamydia PCR test results for patients with negative urinalysis and urine culture results.

Characteristics	Univariate analysis		Multivariate analysis	
	Odds ratio (95% CI)	p value	Odds ratio (95% CI)	p value
Age (years)				
> 30	(Ref)		(Ref)	
< 30	1.83 (0.96, 3.48)	0.065	1.08 (0.44, 2.66)	0.871
Unprotected sex				
No	(Ref)		(Ref)	
Yes	1.71 (0.89, 3.30)	0.109	0.914 (0.36, 2.34)	0.851
Discharge				
No	(Ref)		(Ref)	
Yes	32.50 (14.15, 74.63)	< 0.001	32.669 (13.67, 78.09)	< 0.001

*Ref* reference, *CI* confidence interval.

Among the 142 young adult males with negative results in urinalysis, urine culture, and PCR, the most common diagnosis was balanitis (29 patients, 20.4%), followed by bladder outlet obstruction (20 patients, 14.1%), prostatitis (12 patients, 8.5%), epididymo-orchitis (8 patients, 5.6%), and varicocele (5 patients, 3.5%). Table 3 shows the treatments administered to these patients with the drug use percentage. Medications such as alpha-blockers (28 patients, 19.7%), non-steroidal anti-inflammatory drugs (24 patients, 16.9%), phenazopyridine (40 patients, 28.2%), and empirical antibiotics (97 patients, 68.3%), including doxycycline, cefadroxil, and levofloxacin, were prescribed on the basis of the clinical assessment. Symptoms subsided after one week of treatment in all these patients and these non-infectious diseases were cured through adequate evaluation and treatment with good outcomes.

**Table 3:** Treatment in patients with triple-negative test results.

Treatments	Total (n = 142)
Alpha blocker	28 (19.7%)
Anticholinergics or beta-3 agonist	8 (5.6%)
NSAID	24 (16.9%)
Phenazopyridine	40 (28.2%)
Empirical antibiotics	97 (68.3%)
Cefadroxil	20 (14.1%)
Levofloxacin	13 (9.1%)
Doxycycline	64 (45.1%)

*NSAID* non-steroidal anti-inflammatory drug.

## Discussion

Complaints of urethritis symptoms, including dysuria, increased urination frequency, and urethral discharge, are common in urology clinics. However, the diagnosis and treatment of this condition may be occasionally challenging. In the present study, 142 (38.9%) of the total 365 patients with urethritis symptoms showed negative results in urinalysis, urine culture, and PCR tests for gonorrhoea and chlamydia. The results further highlighted the high frequency of negative urinalysis and urine culture results in young male patients with gonorrhoea- and chlamydia-related urethritis and indicated the importance of urethral discharge (odds ratio: 32.6, p < 0.001) as an indicator for antibiotic treatment of gonorrhoea and chlamydia in these patients. In the triple-negative group, balanitis was the most common tentative diagnosis, and the symptoms subsided in these patients after one-week of empirical treatment. These findings will be of value for urologists treating young males with urethritis symptoms, and the insights provided in this study will facilitate appropriate management and patient satisfaction in such cases.

These patients came to our clinic for urethritis-like symptoms, and gram stain of the urethral secretions could be a rapid diagnostic test to confirm the diagnosis of urethritis^10,11^. However, many of the patients were negative for pyuria on urinalysis, which means that few polymorphonuclear leukocytes (PMNs) could be identified from the Gram stain smear. *N. gonorrhoeae*, a pathogen frequently associated or inside PMNs, may not be necessarily identified from Gram stain smear. Similarly, *C. trachomatis* is also an intracellular pathogen, and may not be seen from Gram stain in patients without discharge at presentation or without pyuria on urinalysis. Besides, the service amount in Taiwan’s urological clinics is usually enormous under the coverage of National Health Insurance. Gram stain done by urologists is time-consuming, and therefore, is not the initial examinations performed by the majority of urologists in Taiwan. Instead of Gram stain, we use urine PCR test at initial exam for patients with urethritis-like symptoms. The convenient sample collection process and quick results provide an effective way of screening multiple pathogens. The study showed that the prevalence of pyuria was 33% and that of positive urine cultures was 21%. For those with pyuria, 69% of which had negative urine culture. A study by Shipman et al. showed similar results, wherein the prevalence of pyuria was 37%, and among the cases with pyuria, 74% had sterile pyuria^3^. Among patients with symptoms of urethritis but negative urinalysis and culture results, up to 30% were diagnosed with gonorrhoea or chlamydia using PCR. Therefore, PCR testing is particularly important in patients with negative urinalysis and urine culture results^5^. However, PCR testing is costly and is not always available in every clinic. In the present study, urethral discharge was reported in 195 (53.4%) of all 365 patients and 183 (82.1%) of 223 patients in the any-positive group. We further compare the urethral discharge differences between PCR positive and negative patients. Of the 139 PCR positive patients, 111 (79.9%) of which presented with urethral discharge, while only 84 (37.2%) of the other 226 PCR negative patients presented with urethral discharge. A study by Bellinato et al. reported urethral discharge in 86% of patients with PCR-identified urethritis^9^. This result is consistent with our findings. Patients with gonococcal urethritis usually present with a purulent discharge, whereas non-gonococcal urethritis is associated with the presence of a transparent discharge^12^. In our study, the presence of discharge was independently associated with PCR positive results for gonorrhoea or chlamydia and could be a guide to prescribe antibiotics against gonorrhoea and chlamydia in clinical practice, in the absence of PCR testing ability.

In the present study, patients with triple-negative tests were significantly older and had less frequent reports of urethral discharge. Most of these patient population have no detectable pathogens. Therefore, we attempted to identify the possible non-infectious aetiologies of urethritis-like symptoms in this patient population. Balanitis was the most common tentative diagnosis in 29 (20.4%) of the 142 triple-negative patients, indicating that physical examination of the genital area is mandatory in patients complaining of urethritis-like symptoms. We arranged further examination to treat these patients based on their clinical judgement. Tentative diagnosis could be prostatic hyperplasia or prostatitis. The pressure flow study and pre-massage and post-massage 2-glass test were obtained, especially in young men. Moreover, all patients reported symptom relief after one week of empirical treatment including empirical antibiotics, phenazopyridine, non-steroidal anti-inflammatory drugs, and alpha-blockers. For young patients with acute-onset urethritis-like symptoms but no pyuria on urinalysis at the first visit, empirical alpha blocker may be used as a symptomatic treatment. If the urine culture or PCR confirmed to have gonococcal or chlamydial infection, treatment would be changed to appropriate antibiotics. For patients with pyuria at first visit, or those with discharge at presentation, empirical antibiotics would be given as the initial treatment instead of alpha blocker. However, we could not determine whether the improvement in symptoms was due to the empirical treatment or if the sterile urethritis-like symptoms were self-limited in our patients. Other studies have also reported that a significant proportion of men with urethritis symptoms have no detectable pathogens^13,14^. Dowd et al. suggested that urethral pain syndrome could be a self-limited illness with a short episode in patients without causality of organisms^15^. Success has been reported in using α-blockers or muscle relaxants as treatment for urethral pain syndrome^16^.

**Figure 2:**
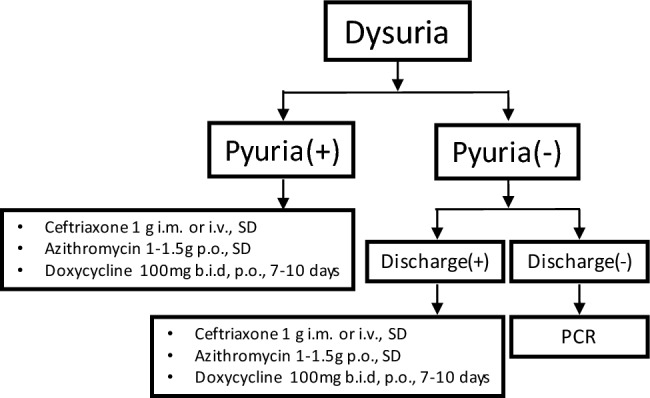
Flowchart of quick clinical diagnosis and treatment for young adult males with dysuria at the first visit.

Based on the findings of the present study, we have provided a flowchart for the management of young male patients with urethritis-like symptoms (Fig. 2). For young patients presenting with positive urinalysis results, we suggest empirical treatment for both *N. gonorrhoeae* and *C. trachomatis*, in accordance with the sexually transmitted disease treatment guidelines^17,18^. Empirical antibiotics including ceftriaxone, azithromycin and doxycycline should be considered in patients with a history of urethral discharge despite of no pyuria on urinalysis at visit. For those without pyuria on urinalysis at visit and no history of urethral discharge, urologists could perform urinary PCR and provide empirical treatments including alpha-blockers, non-steroidal anti-inflammatory drugs or phenazopyridine. The similar empirical treatments are also recommended for patients showing negative urinalysis results but with urethral discharge. The treatment could be tailored depending on the results of urine culture or urinary PCR, as well as the treatment response on the return visit. In the present study, the prognosis of urethritis-like symptoms was quite good even when a pathogen could not be identified. No antibiotic resistance was identified in patients with *N. gonorrhoeae* infection. The study showed that 343 (94%) of the total patients were clinically cured on the first return visit, and no symptom recurrence was noted at the one-month follow-up. Only 22 (6%) of patients complained of persistent discomfort after initial empirical treatment. In another retrospective cohort study, 91.5% and 6.5% of the patients presenting with urethral discharge syndrome were considered clinically cured at the first and second visits, respectively^18^. In addition to medical treatment, reassurance and emotional support may be helpful in improving anxiety associated with urethritis-like symptoms.

This study had some limitations that require consideration. First, it was conducted at a single community hospital, potentially leading to a bias in the prevalence and distribution of microorganisms as well as their resistance to antibiotics. Second, the prevalence of unprotected sexual intercourse may have been underestimated in the present study, which is common in clinical practice. Third, other than chlamydia and gonorrhoea, urethritis pathogens of STIs, such as *M. genitalium*, *U. urealyticum*, and *T. vaginalis*, were not further evaluated in this study. In some cases, patients with polymicrobial NGU may be asymptomatic and the presence of polymicrobial urethritis should be taken into consideration when planning treatment for urethritis^19^. Although the specificity of PCR test is greater than 99%, there is still a potential for a false-positive result. However, the present study will be valuable to urologists practicing. We have provided a simple flowchart for real-time and cost-effective management of young adult males presenting with symptoms of urethritis. We believe that the results of the present study can help prevent the spread of STIs, avoid unwanted complications such as urethral stricture or arthritis, and improve satisfaction in patients with urethritis.

## Conclusions

Infection and non-infectious diseases can cause similar urethritis symptoms in young male patients. Among patients showing negative urinalysis and urine culture results, there is a high frequency of urethral *N. gonorrhoeae* and *C. trachomatis* infections, and the presence of urethral discharge was an independent predictor. Empirical antibiotic treatment is recommended for patients showing positive or negative urinalysis results but with urethral discharge. The prognosis of urethritis-like symptoms was quite good, even when a pathogen could not be identified. Urologists should provide empirical treatment based on their clinical judgment and help relieve their anxiety through reassurance.

## Data Availability

The datasets used and/or analysed during the current study are available from the corresponding author on reasonable request.

## Abbreviations

PCR: Polymerase chain reaction
STI: Sexually transmitted infection
NGU: Non-gonococcal urethritis
PMNs: Polymorphonuclear leukocytes

## References

[1] Bachmann LH (2015). Advances in the understanding and treatment of male urethritis. Clin. Infect. Dis..

[2] Björnelius E (2008). Antibiotic treatment of symptomatic *Mycoplasma genitalium* infection in Scandinavia: A controlled clinical trial. Sex. Transm. Infect..

[3] Shipman SB, Risinger CR, Evans CM, Gilbertson CD, Hogan DE (2018). High prevalence of Sterile Pyuria in the setting of sexually transmitted infection in women presenting to an emergency department. West J. Emerg. Med..

[4] Cook RL, Hutchison SL, Østergaard L, Braithwaite RS, Ness RB (2005). Systematic review: Noninvasive testing for Chlamydia trachomatis and Neisseria gonorrhoeae. Ann. Intern. Med..

[5] Recommendations for the laboratory-based detection of Chlamydia trachomatis and Neisseria gonorrhoeae—2014. *MMWR Recomm. Rep.* **63**, 1–19 (2014). PMC4047970 24622331

[6] Dreger NM, Degener S, Roth S, Brandt AS, Lazica DA (2015). Das Urethralsyndrom: Fakt oder Fiktion—ein Update. Der Urologe.

[7] Kaur, H. & Arunkalaivanan, A. S. Urethral pain syndrome and its management. *Obstetr. Gynecol. Survey* **62** (2007). 10.1097/01.ogx.0000261645.12099.2a 17425813

[8] Van Der Pol B (2021). Clinical performance of the BD CTGCTV2 assay for the BD MAX system for detection of chlamydia trachomatis, neisseria gonorrhoeae, and trichomonas vaginalis infections. Sex. Transm. Dis..

[9] Bellinato, F., Maurelli, M., Gisondi, P., Lleo Fernandez, M. & Girolomoni, G. Clinical profile and co-infections of urethritis in males. *Ital. J. Dermatol. Venerol.* **156**, 681–685. 10.23736/s2784-8671.20.06773-5 (2021). 10.23736/S2784-8671.20.06773-5 33423450

[10] Sarier, M. *et al.* New approach to microscopy of gram-stained urethral smear: The kissing slide method. *Sex. Transm. Dis.* **47** (2020). 10.1097/OLQ.0000000000001228 32649578

[11] Sarier M (2022). Evaluating the utility of the AF Genital System test for pathogen diagnosis in acute male urethritis. Andrologia.

[12] Crotchfelt KA, Welsh LE, DeBonville D, Rosenstraus M, Quinn TC (1997). Detection of Neisseria gonorrhoeae and Chlamydia trachomatis in genitourinary specimens from men and women by a coamplification PCR assay. J. Clin. Microbiol..

[13] Bradshaw CS (2006). Etiologies of nongonococcal urethritis: Bacteria, viruses, and the association with orogenital exposure. J. Infect. Dis..

[14] Schwebke JR (2011). Re-evaluating the treatment of nongonococcal urethritis: Emphasizing emerging pathogens—a randomized clinical trial. Clin. Infect. Dis..

[15] Dowd, T. C. *et al.* Urethral syndrome: a self limiting illness. *Br. Med. J. (Clin. Res. ed.)* **288**, 1349. 10.1136/bmj.288.6427.1349 (1984). 10.1136/bmj.288.6427.1349 PMC1440998 6424854

[16] Phillip H, Okewole I, Chilaka V (2014). Enigma of urethral pain syndrome: Why are there so many ascribed etiologies and therapeutic approaches?. Int. J. Urol..

[17] Workowski KA, Bolan GA (2015). Sexually transmitted diseases treatment guidelines, 2015. MMWR Recomm. Rep..

[18] Menezes Filho, J. R., Sardinha, J. C. G., Galbán, E., Saraceni, V. & Talhari, C. Effectiveness of syndromic management for male patients with urethral discharge symptoms in Amazonas, Brazil. *An. Bras. Dermatol.* **92**, 779–784. 10.1590/abd1806-4841.20175453 (2017). 10.1590/abd1806-4841.20175453 PMC5786390 29364432

[19] Sarier M (2019). Prevalence of polymicrobial infection in urethritis..

